# The Mechanism of Bisphenol A Atherogenicity Involves Apolipoprotein A-I Downregulation through NF-κB Activation

**DOI:** 10.3390/ijms20246281

**Published:** 2019-12-12

**Authors:** Violeta G. Trusca, Madalina Dumitrescu, Ioana M. Fenyo, Irina F. Tudorache, Maya Simionescu, Anca V. Gafencu

**Affiliations:** Institute of Cellular Biology and Pathology “Nicolae Simionescu”, 050568 Bucharest, Romania; violeta.trusca@icbp.ro (V.G.T.); madalina.dumitrescu@icbp.ro (M.D.); madalina.fenyo@icbp.ro (I.M.F.); irina.grosu@icbp.ro (I.F.T.); maya.simionescu@icbp.ro (M.S.)

**Keywords:** apoA-I, apolipoprotein, bisphenol A, NF-κB, promoter, hepatocytes

## Abstract

Apolipoprotein A-I (apoA-I) is the major protein component of high-density lipoproteins (HDL), mediating many of its atheroprotective properties. Increasing data reveal the pro-atherogenic effects of bisphenol A (BPA), one of the most prevalent environmental chemicals. In this study, we investigated the mechanisms by which BPA exerts pro-atherogenic effects. For this, LDLR^−/−^ mice were fed with a high-fat diet and treated with 50 µg BPA/kg body weight by gavage. After two months of treatment, the area of atherosclerotic lesions in the aorta, triglycerides and total cholesterol levels were significantly increased, while HDL-cholesterol was decreased in BPA-treated LDLR^−/−^ mice as compared to control mice. Real-Time PCR data showed that BPA treatment decreased hepatic apoA-I expression. BPA downregulated the activity of the apoA-I promoter in a dose-dependent manner. This inhibitory effect was mediated by MEKK1/NF-κB signaling pathways. Transfection experiments using apoA-I promoter deletion mutants, chromatin immunoprecipitation, and protein-DNA interaction assays demonstrated that treatment of hepatocytes with BPA induced NF-κB signaling and thus the recruitment of p65/50 proteins to the multiple NF-κB binding sites located in the apoA-I promoter. In conclusion, BPA exerts pro-atherogenic effects downregulating apoA-I by MEKK1 signaling and NF-κB activation in hepatocytes.

## 1. Introduction

Apolipoprotein A-I (ApoA-I) is the main structural and functional protein in high-density lipoproteins (HDL) particles, representing approximately 70% of total HDL proteins [1]. ApoA-I comprises eight alpha-helical amphipathic domains of 22 amino acids and two repeats of 11 amino acids and lacks glycosylation or disulfide linkages. Initially, it was proposed that the α-helices of apoA-I are arranged around the circumference of discoidal HDL particles in the so-called “double belt model”, but later other structural models of recombinant discoidal HDL were proposed, such as the “looped belt”, the “solar flares”, and the “double superhelix” model, as reviewed in [2]. Recently, it was revealed that the apoA-I rings on nascent discoidal HDL particles can adopt at least two helical orientations, influencing the activation of lecithin cholesterol acyltransferase (LCAT) in HDL [3]. ApoA-I is more than a structural scaffold that maintains lipid packaging, as apoA-I plays an important role in the transport of cellular cholesterol from the artery wall to the liver for catabolism and acts as a cofactor for LCAT, an enzyme responsible for the formation of most plasma cholesteryl esters [4]. Lack of apoA-I augmented atherosclerosis in hypercholesterolemic mice expressing human apoB [5] and in apoA-I^−/−^/LDLR^−/−^ mice [6]. On the other hand, overexpression of human apoA-I reduced atherogenesis in apoE^−/−^ or in LDLR^−/−^ mice [7,8,9,10,11,12,13,14], providing strong evidence for the antiatherogenic role of apoA-I. Recently, it was proposed that the ratio of HDL-cholesterol to apoA-I gives additional insights as a risk marker for cardiovascular disease [15]. To exploit the anti-atherogenic properties of HDL-apoA-I, different approaches, such as pharmacological interventions using HDL-apoA-I mimetic peptides or infusions of apoA-I-containing particles were proposed for the reduction of atherogenesis [16,17].

In humans, the *APOAI* gene is 1.8 Kb in length and it is located on chromosome 11 in the *APOAI/APOCIII/APOAIV* gene cluster [18]. ApoA-I is synthesized mainly by the liver and the small intestine. *APOAI* gene expression is mainly regulated at transcriptional level [19]. The *APOAI* gene promoter contains a TATA box and several cis elements that regulate its gene expression in either a positive or negative manner. Acidosis caused apoA-I downregulation by promoting binding of repressor proteins to a pH-responsive element that overlaps the TATA box within the apoA-I promoter [20]. Several hormones, such as glucocorticoids, thyroid hormones, and insulin, induced *APOAI* gene expression through a direct mechanism interacting with their specific hormone response elements [21,22]. In humans, treatment with fibrates that interact with a PPAR-responsive element located in the apoA-I promoter increased *APOAI* gene expression, but opposite effects of fibrates on apoA-I expression were found in rodents due to different regulatory elements [23]. Aspirin reduced apoA-I expression at the transcriptional level in HepG2 cells and this effect was reported to be independent of COX inhibition [24]. Several transcription repressors, such as apoA-I repressor protein (ARP-1) and hepatocyte nuclear factor 4 (HNF4) were reported to suppress *APOAI* gene transcription [21]. Regulatory elements and transcription factors that control *APOAI* gene expression are reviewed in [19].

Bisphenol A (2,2-bis [4-hydroxyphenyl]propane, BPA) is one of the common environmental chemicals. Because it is ubiquitously used in the manufacturing of consumer products, human exposure to BPA occurs through beverages and food from BPA-based polycarbonate plastics, but also contaminated water, soil, and air [25,26]. Following oral ingestion, BPA is metabolized in the liver and the intestine into its main metabolite, BPA-monoglucuronide, being removed from the blood by the kidneys in several hours [27]. However, BPA was commonly found in human blood with concentrations between 0.1–100 nM [28] and its conjugated form was found in urines samples with average values between 2–650 nM [29]. An interesting study found a statistically higher BPA concentration in urine samples in females than in males and higher BPA in children than in adults [30], but the reasons are not clear yet. A randomized clinical trial showed that consuming canned beverage augmented urinary BPA concentration and increased blood pressure [31,32]. Prospective studies associated high circulating BPA levels with carotid atherosclerosis [33]. Likewise, cumulative data suggested a link between BPA and cardiovascular disorders and chronic diseases [34,35], but the causal relationship and the underlying mechanisms are unclear. Studies using various strains of transgenic mice and rabbits revealed that BPA aggravates atherosclerosis [36,37,38,39,40]. Numerous studies in rodents evidenced that BPA exposure induces the generation of reactive oxygen species and causes hepatotoxicity [41,42,43,44]. Moreover, BPA induced hepato-steatosis and triggered fatty acids and triglyceride accumulation in HHL-5 hepatocytes [45]. Doses of BPA below the “no observed adverse effect level” promoted oxidative stress and mitochondrial dysfunction in the liver both in vivo and in vitro [46]. The growing evidence regarding the detrimental effects of BPA, such as oxidative stress, inflammation, endocrine disruption, or epigenetic changes [47], makes BPA a major public problem whose solution can be found only after understanding its mechanisms of action.

Our hypothesis states that the pro-atherogenic effects of BPA are mediated, at least in part, by the downregulation of the *APOAI* gene, thus, leading to a decreased amount of the anti-atherosclerotic HDL. This study aimed to investigate whether BPA modulates *APOAI* gene expression and to identify the molecular mechanisms involved in this regulation. We report here that BPA increased the area of the atherosclerotic lesions in the aortas of BPA-treated LDLR^−/−^ mice corroborated with a decrease in HDL and apoA-I levels and an increase in cholesterol and triglycerides plasma concentrations. The mechanism of this process involves the activation of NF-κB pathways and consequently the reduction of apoA-I expression in the liver. Our data demonstrated that the apoA-I promoter contains functional NF-κB binding sites that are responsible for the BPA-induced apoA-I downregulation.

## 2. Results

### 2.1. BPA Increases Plasma Lipid Levels and Atherosclerotic Lesions in LDLR^−/−^ Mice

**Figure 1:**
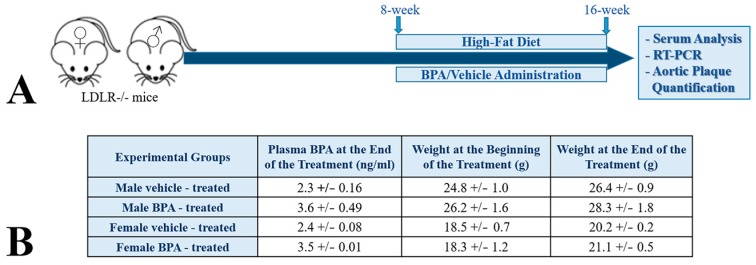
Experimental design and plasma BPA level in the treated mice. (**A**) LDLR^−/−^ mice were fed a high-fat diet and treated with 50 µg BPA/kg body weight by gavage. After two months of BPA treatment combined with the high-fat diet, serum analysis of the lipid levels, hepatic *APOAI* gene expression, and atherosclerotic lesions were evaluated. (**B**) Three days after the last gavage, the plasma BPA concentration was determined. The data showed that in BPA-treated mice the BPA level was slightly increased as compared with the control group. There were no significant differences in body weights of control and BPA-treated animals following exposure to BPA in males or in females.

To test the effect of BPA on lipid metabolism and its role in developing atherosclerosis, eight-week old LDLR^−/−^ mice were fed a high-fat diet and treated with 50 µg BPA/kg body weight, by gavage, as illustrated in the scheme in Figure 1A. We analyzed the pro-atherogenic effects of BPA in males as well as in females LDLR^−/−^ mice because in females BPA may act also as an endocrine disrupter and may aggravate atherogenesis [36,37,38,39,40]. As shown in Figure 1B, at the end of the two months of treatment, plasma BPA levels were slightly increased, in a similar manner in males and females LDLR^−/−^ mice (Figure 1). The body weight of the BPA-treated mice (measured at the beginning and at the end of treatment) did not significantly increase as compared to those of the control group (Figure 1B).

**Figure 2:**
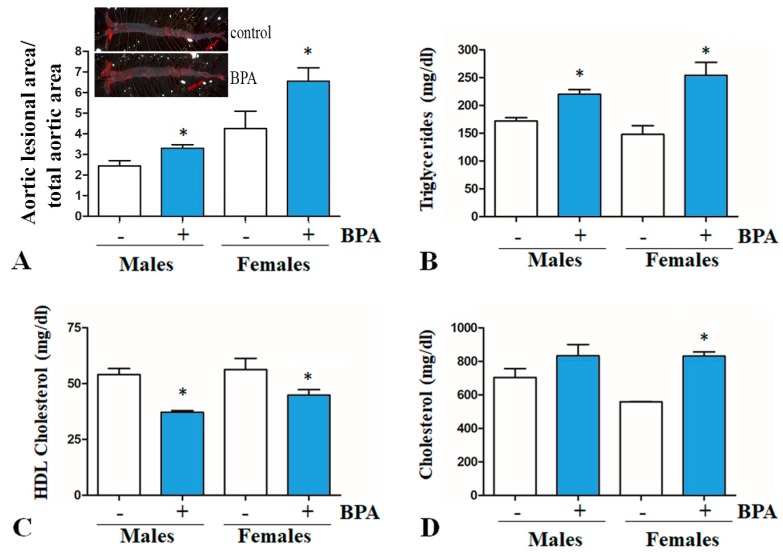
Aortic atherosclerotic lesions quantification and plasma lipids in LDLR^−/−^ mice exposed to BPA. (**A**) Area of aortic atherosclerotic lesions of the BPA-treated LDLR^−/−^ mice was significantly higher as compared to those in the control group, in which only vehicle (water) was administered (*p* < 0.05). Representative images of aortas stained with Oil red O in females LDLR^−/−^ mice are presented on top of Panel A. (**B**) Plasma triglycerides levels in BPA-treated mice were significantly higher compared to those in the control group for both genders (* *p* < 0.05). (**C**) In the BPA-treated mice group, HDL-cholesterol levels were significantly lower as compared to those in the control group for males and females (* *p* < 0.05). (**D**) Cholesterol levels were significantly increased in the BPA-treated female mice as compared to the control group; for males, an increase was registered but it was not statistically significant (* *p* > 0.05).

After two months of BPA-treatment, the area of atherosclerotic lesions in the aorta (Figure 2A) and triglycerides concentrations (Figure 2B) were significantly increased, while HDL-cholesterol was decreased (Figure 2C) in BPA-treated LDLR^−/−^ mice as compared with the control group, in females as well as in males. In BPA-treated female mice, total cholesterol levels were significantly higher as compared to those in the control group; for BPA-treated male mice, the cholesterol levels were higher but not statistically significant (Figure 2D).

### 2.2. BPA Downregulates apoA-I in Hepatocytes

**Figure 3:**
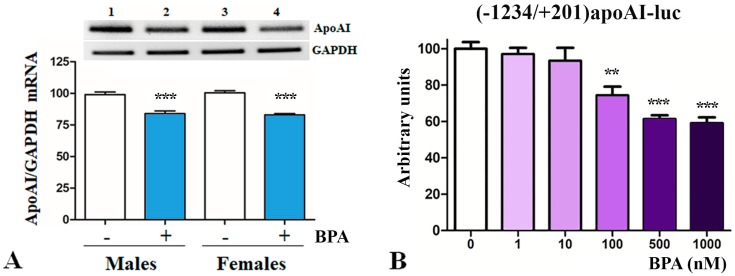
Down-regulatory effect of BPA on apoA-I in hepatocytes. (**A**) Real-Time PCR data showed that BPA treatment of LDLR^−/−^ mice significantly decreased hepatic apoA-I mRNA levels as compared to control mice, as depicted in the graph. Representative images illustrate the expression of apoA-I and GAPDH genes in the liver, as detected by RT-PCR. (**B**) Transiently transfected HepG2 cells with [−1234/+201]apoAI-luc construct were treated with BPA (1–1000 nM) and the activity of apoA-I promoter was assessed by luciferase assays. BPA treatment with low concentrations (1 and 10 nM) had no effect on apoA-I promoter activity, while higher concentrations (100–1000 nM) decreased the activity of apoA-I promoter. Statistical significance ** *p* < 0.01, *** *p* < 0.005.

Due to the decrease in HDL-cholesterol levels observed under BPA-treatment, next we tested apoA-I expression in the liver. The hepatic apoA-I expression was evaluated by Reverse Transcription-Polymerase Chain Reaction (RT-PCR) and quantified by Real-Time PCR. ApoA-I expression was normalized to GAPDH gene expression. Data showed that BPA-treatment significantly decreased hepatic apoA-I mRNA levels in LDLR^−/−^ mice as compared to control mice (*p* < 0.001, Figure 3A). Representative images of Midori-stained agarose gels from RT-PCR experiments showing hepatic apoA-I and GAPDH gene expression in liver isolated from BPA-treated and control mice are presented on top of Figure 3A.

Next, we assessed the capacity of BPA to modulate the transcriptional activity of apoA-I promoter, by transfection experiments in hepatocytes. For this, HepG2 cells were transiently transfected with the [−1234/+201]apoAI-luc construct, and after 18 h, cells were treated with increasing concentrations of BPA (1–1000 nM). After 36 h after transfection, the activity of apoA-I promoter was evaluated based on the luciferase reporter gene and normalized to the co-transfected β-galactosidase. As illustrated in Figure 3B, we found that BPA inhibits the apoA-I promoter activity in a dose-dependent manner. The treatment of HepG2 cells with 500–1000 nM BPA caused a significant reduction of the activity of apoA-I promoter down to ~60% of the control value (*p* = 0.0011), while 100 nM BPA decreased the activity of apoA-I promoter to ~74% of the control value (*p* = 0.013). The treatment of hepatocytes with low concentrations of BPA (1 nM or 10 nM BPA) did not significantly affect the apoA-I promoter activity (Figure 3B).

### 2.3. The Mechanisms of apoA-I Downregulation by BPA

**Figure 4:**
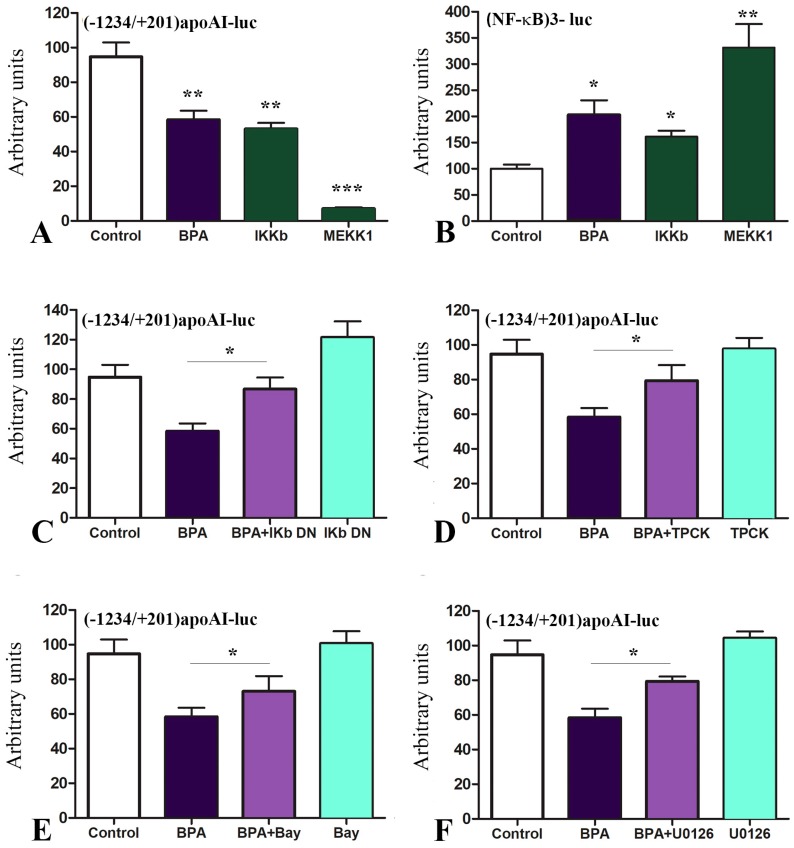
BPA downregulates apoA-I promoter activity through NF-κB activation. (**A**) HepG2 cells were transfected with the [−1234/+201]apoAI-luc construct and treated with BPA or were simultaneously transfected with overexpression vectors for IKKB or MEKK1. BPA decreased apoA-I promoter activity in a similar manner as IKKB overexpression. MEKK1 overexpression strongly reduced the apoA-I promoter activity down to ~7% of the control value. (**B**) When HepG2 cells were transfected with the construct (NF-κB)_3_-luc, BPA increased the promoter activity, similarly with the overexpression of IKKB and MEKK1—the specific inducers of this promoter. (**C**–**E**) When HepG2 cells transfected with [−1234/+201]apoAI-luc were treated with NF-κB specific inhibitors, apoA-I promoter activity downregulation induced by BPA was reduced. The treatment with the inhibitors alone did not affect the promoter activity. (**F**) The treatment with U0126 (MEKK1 inhibitor) of the cells transfected with [−1234/+201]apoAI-luc construct led to the reversion of the apoA-I promoter inhibition by BPA. U0126 alone did not affect the apoA-I promoter activity in transfected cells. Statistical significance * *p* < 0.05, ** *p* < 0.01, *** *p* < 0.005.

Next, we searched for the mechanisms underlying the downregulation of apoA-I by BPA and tested whether BPA exerts its downregulatory effect on apoA-I through mitogen-activated protein kinase kinase kinase 1 (MEKK1) and NF-κB activation. For this purpose, we transiently transfected HepG2 cells with the [−1234/+201]apoAI-luc construct simultaneously with overexpression of IκB kinase (IKKB) or MEKK1 or the transfected cells were treated with BPA (1000 nM). Our data illustrated in Figure 4A showed that the treatment of hepatocytes with BPA caused a reduction in the activity of apoA-I promoter down to ~60% of the control value (*p* = 0.001). IKKB overexpression decreased the apoA-I promoter activity down to ~53% (*p* = 0.009), while MEKK1 overexpression drastically decreased the apoA-I promoter activity down to ~7% of the control value (*p* = 0.0004) (Figure 4A).

In parallel, we assessed the capacity of BPA to activate a promoter containing NF-κB binding sites (Figure 4B). For this purpose, we transiently transfected hepatocytes with the construct (NF-κB)_3_-luc containing three copies of the NF-κB binding site cloned upstream of the minimal SV40 promoter and the luciferase reporter gene in pGL2-Promoter Vector (Promega). Treatment of hepatocytes with BPA increased ~2.4-fold the activity of the construct (NF-κB)_3_-luc (*p* < 0.01), as presented in Figure 4B. As a control, we overexpressed IKKB or MEKK1 together with the construct (NF-κB)_3_-luc in HepG2 cells and we obtained an increase in the luciferase activity (Figure 4B).

Next, we searched if NF-κB inhibitors could revert BPA downregulation of apoA-I. For this, cells were transfected with [−1234/+201]apoAI-luc construct simultaneously with the overexpression of IKb dominant-negative (IKb DN) (Figure 4C). In other experiments, after 24 h from transfection with the aforementioned construct, the transfected cells were treated with NF-κB inhibitors tosyl phenylalanyl chloromethyl ketone (TPCK), Bay 11-7082, or a MEK1/2 inhibitor (U0126). Our results showed that NF-κB inhibitors (TPCK and Bay 11-7082), IKb DN or U0126 (MEK1/2 inhibitor) significantly reverted the downregulatory effect of BPA on apoA-I promoter activity. The treatment of HepG2 cells with the inhibitors alone (Bay 11-7082, TPCK, IKb DN or U0126) did not significantly affect the apoA-I promoter activity (Figure 4C–F).

**Figure 5:**
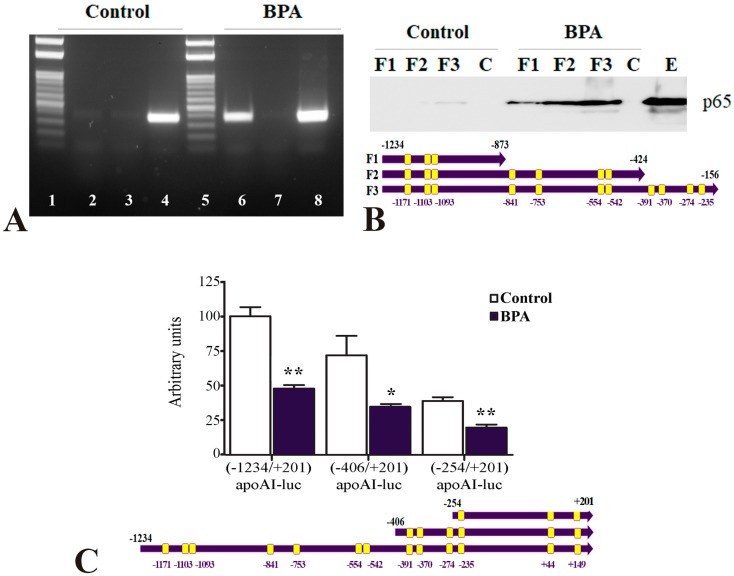
NF-κB binds to apoA-I promoter in BPA-treated hepatocytes. (**A**) Chromatin immunoprecipitation assays showed that p65/50 proteins are recruited to the apoA-I promoter in BPA-treated HepG2 cells (lane 6). No binding of p65/50 proteins was detected when the cells were not exposed to BPA (lane 2) or when IgG antibodies were used in chromatin immunoprecipitation (lanes 3 and 7). PCR using the input prepared from BPA-treated or untreated hepatocytes as a template gave the expected product (lanes 4 and 8). The DNA ladder is represented in lanes 1 and 5. (**B**) DNA pull-down assays showed that p65/50 proteins bound efficiently to the three biotinylated 3′-deletion mutants of the apoA-I promoter in BPA-treated hepatocytes (BPA: F1, F2, and F3), illustrated below the graph. In untreated hepatocytes, no binding was recorded for −1234/−873 and −1234/−424 fragments of apoA-I promoter fragments (Control: F1 and F2) and a very faint band showed that p65/50 proteins weakly bound to the whole apoA-I promoter (F3, −1234/−156). No binding of p65/50 was observed to biotinylated DNA used as a negative control (Control-C or BPA-C). Western blot of p65 in the nuclear extract isolated from untreated HepG2 cells is represented in the last lane, E. (**C**) HepG2 cells were transfected with 5′-deletion mutants: [−1234/+201]apoA-I-luc, [−406/+201]apoAI-luc and [−254/+201]apoAI-luc (illustrated below the graph) treated with 1 µM BPA. BPA significantly decreased the activity of all the apoA-I promoter fragments tested. Statistical significance * *p* < 0.05, ** *p* < 0.01.

Next, we revealed the NF-κB binding on apoA-I promoter in hepatocytes (BPA-treated HepG2) by chromatin immunoprecipitation experiments. For this, the cross-linked chromatin isolated from BPA-treated hepatocytes was immunoprecipitated using anti-p65 antibodies and PCR was performed using primers that amplified the region −1221/−870 of the human apoA-I promoter (presented in Table 1). Our results indicated that p65/50 proteins are recruited to the apoA-I promoter in BPA-treated HepG2 cells (Figure 5A, lane 6). No binding of p65/50 proteins was detected when the cells were not exposed to BPA (Figure 5A, lane 2) or when IgG antibodies were used in chromatin immunoprecipitation (Figure 5A, lanes 3 and 7). PCR using the input prepared from BPA-treated or untreated hepatocytes as a template gave the expected product (Figure 5A, lanes 4 and 8). The DNA ladder is represented in the lanes 1 and 5 of Figure 5A.

To identify the region of the apoA-I promoter required for downregulation by BPA, we searched for the putative NF-κB binding sites. In silico sequence analysis [48] predicted the presence of 13 NF-κB binding sites in the apoA-I promoter, as schematically illustrated in Figure 5C. We investigated whether these potential binding sites for NF-κB in the apoA-I promoter are functional in hepatocytes. For this purpose, we performed DNA pull-down experiments using nuclear extracts prepared from BPA-treated or untreated HepG2 cells and biotinylated DNA corresponding to the following three regions of the apoA-I promoter: −1234/−873 (F1), −1234/−424 (F2), −1234/−156 (F3). The results of DNA pull-down assays showed that in BPA-treated hepatocytes, p65/50 proteins bound efficiently to all the three biotinylated DNA fragments of apoA-I promoter (Figure 5B, BPA: F1, F2, and F3). It is worth to mention that higher amounts of p65/50 proteins bound on the −1234/−156 apoA-I promoter fragment (containing 11 p65/50 binding sites) as compared to the other two smaller fragments of apoA-I promoter (containing three or seven p65/50 binding sites). In untreated hepatocytes, no binding was detected to the −1234/−873 and −1234/−424 apoA-I promoter fragments (Figure 5B, Control: F1 and F2) and a faint band indicated that p65/50 proteins weakly bound to the −1234/−156 fragment of apoA-I promoter (Figure 5B, Control: F3). No binding of p65/50 to biotinylated unspecific DNA used as a negative control was observed neither in untreated nor in BPA-treated cells (Figure 5B, Control-C and BPA-C). Western blot of p65 in the nuclear extract isolated from HepG2 cells is represented in lane E of Figure 5B.

Next, we constructed two deletion mutants of the apoA-I promoter to eliminate several NF-κB binding sites and to study their role in the inhibitory effect of BPA on apoA-I promoter activity. Transfected HepG2 cells with the deletion mutants ([−1234/+201]apoAI-luc, [−406/+201]apoAI-luc, and [−254/+201]apoAI-luc) were treated with BPA (1 µM). Data illustrated in Figure 5C showed that BPA treatment significantly decreased the activity of all three apoA-I promoter fragments that were tested, a result revealing the potency of the NF-κB binding sites found at −254 in the apoA-I proximal promoter or in the first exon.

Taken together, the results of transient transfections, DNA pull-down assays and ChIP experiments indicate that treatment of hepatocytes with BPA promotes NF-κB binding to apoA-I promoter.

## 3. Discussion

The anti-atherosclerotic protein apoA-I is the most abundant protein component of HDL particles (~70 % of total HDL proteins), which promotes cholesterol efflux from tissues to the liver for excretion [49,50]. The main sites for apoA-I biosynthesis and secretion are the liver and the small intestine. Reduced hepatic apoA-I expression by high cytokines levels was reported in diabetes [51]. Data from the literature showed that inflammatory stress (IL1-β and TNFα) down-regulated apoA-I expression in hepatocytes [52,53].

Bisphenol A (2,2-bis[4-hydroxyphenyl]propane, BPA) is one of the most prevalent environmental chemicals. Following ingestion, BPA is metabolized in the liver and intestine with an extensive formation of BPA monoglucuronide, which is supposed to be eliminated in the urine in several hours [54]. However, there are numerous studies that associated BPA exposure with liver toxicity, production of reactive oxygen species, increased blood pressure, cardiovascular disorders, obesity, diabetes, and reproductive problems [55]. Many data regarding the pathological effects of BPA came from studies using animal models of atherosclerosis, but there are still limited mechanistic details. There is growing evidence suggesting that BPA exacerbates atherosclerosis [36,37,38,39,40]. Our results are in agreement with this data and showed that BPA treatment aggravates atherosclerosis in male and female LDLR^−/−^ mice (Figure 2A). In BPA-treated mice, the level of triglycerides increased (Figure 2B), while plasma HDL-cholesterol concentration decreased (Figure 2C) as compared to control mice. Prompted by the decrease of HDL-cholesterol level by BPA treatment, we evaluated the hepatic apoA-I expression in BPA-treated LDLR^−/−^ mice. Real-Time PCR data indicated reduced hepatic apoA-I mRNA levels in BPA-treated mice as compared to controls (Figure 3A). Moreover, our data showed that in HepG2 cells BPA inhibits the activity of apoA-I promoter in a dose-dependent manner (Figure 3B). These results are in agreement with a previous study that reported accumulation of triglycerides and total cholesterol, decreased HDL-cholesterol levels, and reduced levels of apolipoproteins apoA-I, apoA-IV, and apoC-II in BPA-treated CD-1 mice (2.5 µg/L BPA in drinking water estimated as 0.5 µg BPA/kg/day) for 10 months [56]. Long-term exposure to BPA induced hypercholesterolemia as well as hyperglycemia in male CD-1 mice [57]. In Fischer 344 female rats, increased liver fat infiltration and plasma apoA-I was reported as a consequence of combined fructose and BPA exposure [58]. In ICR female mice that were prenatally exposed to BPA in drinking water, apoA-I expression was found to be increased in the immune organs, thymus, and spleen, in a dose-dependent manner [59]. Once we established that BPA decreases apoA-I expression in both males and females LDLR^−/−^ mice, we tested whether the hepatic apoA-I down-regulation by BPA is a transcriptional process. 

Data from the literature revealed that BPA promoted the generation of pro-inflammatory cytokines, inducing the phosphorylation and nuclear translocation of NF-κB p65 [60]. Low doses of BPA interfere with insulin signaling pathways, impairing energy and glucose metabolism in adipose cells, a process associated with an increase in IL-6 and IFN-γ secretion and activation of JNK, STAT3, and NF-κB pathways [61]. In addition, it was shown that NF-κB was implicated in lipopolysaccharide (LPS) down-regulation of apoA-I promoter activity in HepG2 cells, a process mediated by PPARα [62]. Other data showed that histamine down-regulated apoA-I in hepatocytes via histamine H1 receptor and increased NF-κB expression, which directly acted on apoA-I promoter, as determined by ChIP assays [63]. However, the exact location of the NF-κB binding sites has not been reported yet. Thus, we further investigated the mechanisms underlying the apoA-I downregulation by BPA in hepatocytes testing whether BPA exerts its inhibitory effects on apoA-I through NF-κB activation. Our data showed that BPA is able to activate NF-κB and to downregulate the apoA-I promoter in a similar manner as IKKB overexpression (Figure 4A,B). This process was specific since it was inhibited by overexpression of IkB dominant-negative or by treatment with NF-κB inhibitors (Figure 4C–E). We also tested the possible implication of MEKK1 and MEK1/2 in BPA downregulation of apoA-I, and we found that both kinases are involved in this process (Figure 4A,F). This mechanism is similar to that reported for BPA induction of IL-6 and TNFα in microglial cells, which involved MEK1/2 signaling and NF-κB [64].

Since the analysis of apoA-I promoter revealed the presence of 13 NF-κB binding sites [48], and the results of ChIP experiments showed that BPA promotes NF-κB recruitment to apoA-I promoter in hepatocytes (Figure 5A), we further tested whether these NF-κB binding sites are responsible for apoA-I downregulation by BPA. The results of DNA pull-down assays and transfection experiments using deletion mutants of apoA-I promoter showed that p65/50 proteins are recruited to the apoA-I promoter after treatment of hepatocytes with BPA (Figure 5B,C). Surprisingly, the inhibitory effect of BPA on apoA-I promoter activity was still recorded for two 5′-deletion mutants of the apoA-I promoter lacking several NF-κB binding sites, suggesting the potency of the NF-κB binding sites found at −254/+201 in the apoA-I promoter. Our data represent the first report regarding the location of the multiple active NF-κB binding sites in the apoA-I promoter.

In conclusion, our findings indicate that BPA aggravates atherosclerosis in LDLR^−/−^ mice. The detrimental effects of BPA are, at least in part, a consequence of NF-κB activation, which downregulates apoA-I gene expression, subsequently leading to a decreased amount of the anti-atherosclerotic HDL particles.

## 4. Materials and Methods

*Chemicals*. Bisphenol A (239658) and Bay 11-7082 were purchased from Sigma-Aldrich (St. Louis, MO, USA), mouse Bisphenol A ELISA Kit (#MBS071884) was from MyBioSource. GoTaq DNA polymerase and Luciferase Assay System were obtained from Promega Corp. (Madison, WI, USA). FastDigest restriction enzymes were from Thermo Scientific. Dulbecco’s Modified Eagle’s Medium (DMEM) and fetal calf serum were purchased from EuroClone (Milano, Italy), and Super Signal West Pico chemiluminescent substrate was from Pierce (Rockford, USA). TRIzol reagent was purchased from Invitrogen Life Technologies (Carlsbad, CA, USA). Dynabeads M-280 streptavidin magnetic beads were from Invitrogen Dynal (Oslo, Norway). Primers were from Microsynth AG (Balgach, Switzerland). Midori Green Advanced DNA Stain (MG-02) was from Nippon Genetics Europe (Germany). Antibodies were from Santa Cruz Biotechnology (Santa Cruz, CA, USA). All other chemicals were from Sigma-Aldrich (St. Louis, MO, USA).

*Animal experimentation*. LDLR^−/−^ mice (Charles River Laboratories, MA, USA) receiving standard rodent diet and water ad libitum were exposed to 12 h cycles of light and dark. For each experiment, 8-weeks old mice were randomly distributed into the following experimental groups: (i) the ‘BPA’ group received Bisphenol A (50 µg/kg body weight in 100 µL water) by gavage three times per week (*n* = 6 animals); (ii) the ‘Control’ group received 100 µL water by gavage (*n* = 5 animals). After two months of BPA treatment and an atherogenic diet (containing 1% cholesterol), the animals were sacrificed. Blood was harvested for lipid analysis; the liver was taken for RNA isolation and the aorta was harvested, fixed in paraformaldehyde (PFA) 4% and stained with Oil Red O for en face evaluation of the atherosclerotic lesions using AxioVision software. The animal experimentation was conducted in accordance with the EU Directive 63/2010 and the European Convention for the Protection of Vertebrate Animals used for Experimental and Other Scientific Purposes, and the protocols were approved by the Ethical Committee at the Institute of Cellular Biology and Pathology “Nicolae Simionescu” and by National Sanitary Veterinary and Food Safety Authority (348/14.04.2017).

*Real-Time PCR*. Total cellular RNA was extracted using TRIzol reagent and reverse transcription was performed with a High-Capacity cDNA reverse transcription kit (Applied Biosystems). Real-Time PCR was performed using primers shown in Table 1 and Syber Green Mix on a 7900 HT Applied Biosystems machine (Applied Biosystems, CA, USA). ApoA-I levels were normalized to GAPDH expression.

Plasmid constructions. Human apoA-I promoter (−1234/+201 apoAI) was cloned in SacI and XhoI sites in the pGL4.14[luc2/Hygro] vector (Promega), which contains the promoterless luciferase (luc) gene as a reporter. ApoA-I promoter sequence was confirmed by sequencing using Forward RV3 and Reverse RV4 primers shown in Table 1. Based on the construct [−1234/+201]apoAI-luc, two 5′-deletion mutants of the apoA-I promoter were constructed. The first 5′-deletion mutant of apoA-I promoter was obtained by PCR amplification using the plasmid [−1234/+201]apoAI-luc as a template and the primers shown in Table 1 (Forward apoA-I promoter-406, Reverse apoA-I promoter+201). The obtained DNA fragment was inserted in pGL4.14[luc2/Hygro] vector in SacI and XhoI sites, resulting in the construct [−406/+201]apoAI-luc. To obtain the second 5′-deletion mutant of apoA-I promoter, the plasmid [−1234/+201]apoAI-luc was cleaved with SacI and SmaI restriction enzymes, T4 DNA polymerase was used to obtain blunt DNA ends and T4 DNA ligase catalyzed the ligation of blunt-ended DNA, resulting in the construct [−254/+201]apoAI-luc.

*Cell culture and transfection.* HepG2 hepatocytes (ATCC, MD, USA) were grown in DMEM with 4.5‰ glucose and 10% fetal bovine serum. HepG2 cells were transiently transfected by the Ca_3_(PO_4_)_2_ precipitation method. When luciferase was used as a reporter gene, its activity was assayed with the Luciferase Assay Kit and transfection efficiency was normalized to β-galactosidase activity as we previously described [65,66,67,68]. The experiments were done in triplicate and repeated three times.

*DNA pull-down assays*. This assay was carried out using nuclear extracts purified from normal or BPA-treated HepG2 cells. Biotinylated DNA (corresponding to the three regions of apoA-I promoter immobilized on Dynabeads M-280 Streptavidin, was used as previously reported [69,70,71]. The sequence of the oligonucleotides used for DNA pull-down assays is shown in Table 1. NF-κB p65/50 proteins bound to the biotinylated oligonucleotides were detected by immunoblotting using anti-p65 antibodies followed by HRP-labeled secondary antibodies. The proteins were revealed using the Super Signal West Pico chemiluminescent substrate.

*Chromatin immunoprecipitation*. Chromatin immunoprecipitation experiments were done using chromatin from HepG2 hepatocytes (control or BPA-treated). Chromatin was immunoprecipitated with anti-p65 antibodies for 18 h at 4 °C. The precipitated DNA sequences were analyzed by PCR using the primers shown in Table 1 and the PCR products (351 bp) were analyzed by agarose gel electrophoresis after staining with Midori Green Advanced DNA Stain.

*Statistics*. Results were expressed as means ± standard deviation. Statistical significance was calculated using a two-tailed *t*-test (GraphPad Prism 5 Software, San Diego, CA, USA). At *p* < 0.05, the population means are significantly different.

## Figures and Tables

**Table 1:** The sequences of the primers used for cloning, Real-Time PCR, DNA pull-down assays and chromatin immunoprecipitation experiments.

**Primers for Human apoA-I Promoter Cloning:**	
Forward apoA-I promoter−1234	5′-AAAGTCCCTCTGTGCTCTTCAGTC
Forward apoA-I promoter−406	5′-GGAATGCTGGTGGTGGGGGAG
Reverse apoA-I promoter+201	5′-AAAACTCGAGGAAGGGCCTGGCTGAGTGGGGTGC
**Primers for Real-Time PCR**	
Mouse apoA-I Forward	5′-CAAGACAGCGGCAGAGAC
Mouse apoA-I Reverse	5′-CACCTTCTGTTTCACTTCC
GAPDH Forward	5′-ACCACAGTCCATGCCATCAC
GAPDH Reverse	5′-TCCACCACCCTGTTGCTGTA
**Primers for DNA Pull-Down Assays**	
RV3 Forward-Biotin	5′-Biotin-CTAGCAAAATAGGCTGTCCC
ApoA-I Reverse-873	5′-GGGCAAAGGAAGTGGCAGGAG
ApoA-I Reverse-424	5′-GACTACTTCACTCCCCTCCCCC
ApoA-I Reverse-156	5′-GGGCAAATAGAGTGGGCAAACAGC
**Primers for Chromatin Immunoprecipitation**	
Forward apoA-I promoter	5′-AAAGTCCCTCTGTGCTCTTCAGTC
Reverse apoA-I promoter	5′-GGGCAAAGGAAGTGGCAGGAG
**Primers for Sequencing**	
RV3 Forward	5′-CTAGCAAAATAGGCTGTCCC
RVprimer4 Reverse	5′-GACGATAGTCATGCCCCGCG

## Abbreviations

apoA-I	apolipoprotein A-I
*APOAI*	apolipoprotein A-I gene
BPA	Bisphenol A
NF-κB	Nuclear Factor Kappa B
JNK	c-Jun N-terminal kinase
MEKK1	Mitogen-activated protein kinase kinase kinase 1
LPS	Lipopolysaccharide
HDL	High Density Lipoprotein
LDL	Low Density Lipoprotein
VLDL	Very Low-Density Lipoprotein
LCAT	Lecithin Cholesterol Acyltransferase
ChIP	Chromatin immunoprecipitation
DNAP	DNA pull-down assays
Luc	Luciferase
HNF4	Hepatocyte nuclear factor 4
RT-PCR	Reverse Transcription-Polymerase Chain Reaction
IKKB	IκB kinase
IKb DN	IKb dominant-negative
DMEM	Dulbecco’s Modified Eagle’s Medium
PFA	Paraformaldehyde
